# Analysis of Pulmonary Inflammation and Function in the Mouse and Baboon after Exposure to *Mycoplasma pneumoniae* CARDS Toxin

**DOI:** 10.1371/journal.pone.0007562

**Published:** 2009-10-27

**Authors:** R. Doug Hardy, Jacqueline J. Coalson, Jay Peters, Adriana Chaparro, Chonnamet Techasaensiri, Angelene M. Cantwell, T. R. Kannan, Joel B. Baseman, Peter H. Dube

**Affiliations:** 1 Department of Microbiology and Immunology, University of Texas Health Sciences Center at San Antonio, San Antonio, Texas, United States of America; 2 Department of Medicine, Division of Pulmonary and Critical Care Medicine, University of Texas Health Sciences Center at San Antonio, San Antonio, Texas, United States of America; 3 Department of Pathology, University of Texas Health Sciences Center at San Antonio, San Antonio, Texas, United States of America; 4 Department of Internal Medicine and Pediatric Infectious Diseases, University of Texas Health Sciences Center at San Antonio, San Antonio, Texas, United States of America; 5 University of Texas Health Sciences Center at San Antonio, San Antonio, Texas, United States of America; 6 University of Texas Southwestern Medical Center, Dallas, Texas, United States of America; University of California Merced, United States of America

## Abstract

*Mycoplasma pneumoniae* produces an ADP-ribosylating and vacuolating toxin known as the CARDS (Community Acquired Respiratory Distress Syndrome) toxin that has been shown to be cytotoxic to mammalian cells in tissue and organ culture. In this study we tested the ability of recombinant CARDS (rCARDS) toxin to elicit changes within the pulmonary compartment in both mice and baboons. Animals responded to a respiratory exposure to rCARDS toxin in a dose and activity-dependent manner by increasing the expression of the pro-inflammatory cytokines IL-1α, 1β, 6, 12, 17, TNF-α and IFN-γ. There was also a dose-dependent increase in several growth factors and chemokines following toxin exposure including KC, IL-8, RANTES, and G-CSF. Increased expression of IFN-γ was observed only in the baboon; otherwise, mice and baboons responded to CARDS toxin in a very similar manner. Introduction of rCARDS toxin to the airways of mice or baboons resulted in a cellular inflammatory response characterized by a dose-dependent early vacuolization and cytotoxicity of the bronchiolar epithelium followed by a robust peribronchial and perivascular lymphocytic infiltration. In mice, rCARDS toxin caused airway hyper-reactivity two days after toxin exposure as well as prolonged airway obstruction. The changes in airway function, cytokine expression, and cellular inflammation correlate temporally and are consistent with what has been reported for *M. pneumoniae* infection. Altogether, these data suggest that the CARDS toxin interacts extensively with the pulmonary compartment and that the CARDS toxin is sufficient to cause prolonged inflammatory responses and airway dysfunction.

## Introduction

*Mycoplasma pneumoniae* is a human pathogen that preferentially infects the respiratory tract causing acute and chronic pulmonary disease [1], [2]. Recent data suggests *M. pneumoniae* is responsible for 20–40% of all community acquired pneumonia, is frequently linked to upper respiratory infections leading to tracheobronchitis and pharyngitis, and is implicated in airway dysfunction, including asthma [3]. However, an accurate estimate of the extent of *M. pneumoniae* infection in the general population is lacking, and infection is probably more extensive than currently accepted due to difficulties in culturing the organism from clinical samples and unreliable diagnostic laboratory tests [4]. Primary *M. pneumoniae* infection can persist for weeks, and data from both humans and animal models of disease suggest that *M. pneumoniae* infection exacerbates chronic respiratory diseases, such as asthma and chronic obstructive pulmonary disease (COPD) [3], [5], [6], [7], [8]. As many as 20–30% of asthma exacerbations have been linked to *M. pneumoniae* infection [5], [6], [7], [8].

Outside of the respiratory tract, *M. pneumoniae* infections have been associated with pathologies of the central nervous system, cardiovascular system, kidneys, pancreas, liver, skin, and hematopoietic compartments [3]. In rare cases, fulminate disease with fatal outcomes is possible (manuscript in preparation).

Pulmonary infection of mice with *M. pneumoniae* leads to a robust inflammatory response characterized by increases in pro-inflammatory cytokines and chemokines, lobular pneumonia, as well as perivascular and peribronchiolar lymphocytic infiltrates [9], [10], [11], [12], [13], [14], [15], [16]. We and others have reported that in mouse models of *M. pneumoniae*-mediated disease these acute inflammatory responses can persist in various forms for at least 35 days [11], [12]. Besides acute inflammatory reactions, chronic inflammatory responses can be observed in animal models of infection. For example, in animals that are sensitized to ova albumin and infected with *M. pneumoniae*, airway remodeling can be observed suggesting that *M. pneumoniae* mediated inflammatory responses are capable of causing long-term lung damage [10]. Recent studies have shown that Toll-like receptors 1, 2, and 6 are important innate immune receptors for the detection of *M. pneumoniae* lipoproteins, leading to the activation of NF-κB and the production of mucin in airway cells [17], [18], [19], [20], [21], [22]. The extent of Toll-like receptor-mediated inflammation in response to *M. pneumoniae* infection is currently unknown, as is the full repertoire of immune-stimulatory antigens produced by this pathogen.

*Mycoplasma pneumoniae* is an atypical bacterium that lacks a cell wall, has one of the smallest genomes known, requires a range of host-derived nutritional components, and uses the UGA codon to encode tryptophan [23]. Despite its limited genome, *M. pneumoniae* possesses numerous virulence-related genes, specifically linked to cytadherence and colonization [23], [24], [25], [26]. However, unlike many bacterial pathogens, *M. pneumoniae* lacks numerous classical virulence determinants, such as specialized virulence-associated secretion systems (type three/four) and their associated secreted virulence products. Recently, Kannan and Baseman reported the identification and initial characterization of the first *M. pneumoniae* toxin termed the CARDS toxin (MPN372) [27], [28].

The CARDS toxin is an ADP-ribosylating and vacuolating toxin with homology to the S1 subunit of pertussis toxin that has a high affinity for surfactant protein-A, suggesting a physiological role for the toxin in the pulmonary compartment [27], [28]. Recombinant CARDS toxin displays a dose-dependent cytotoxic effect on both tissue culture cells and baboon tracheal epithelium, consistent with what is observed during *M. pneumoniae* infection [27]. Not surprisingly, the cytotoxic effect of CARDS toxin is dependent on the enzymatic activity of the toxin because heat-inactivated (HI) toxin has no effect on mammalian cells or tissues in culture [27]. A number of bacterial pathogens produce ADP-ribosylating or vacuolating toxins that contribute to the pathogenesis of diseases through the induction of inflammation, but the CARDS toxin is the first example of a toxin that exhibits both ADP-ribosylating and vacuolating properties [27], [29]. Until now, it was unknown if the CARDS toxin directly impacted on the pathogenesis of *M. pneumoniae* infection or the extent of host inflammatory responsiveness.

Here we report that rCARDS toxin is a potent inducer of pulmonary inflammation in mice and baboons. rCARDS toxin-mediated inflammatory responses are characterized by the rapid expression of cytokines and chemokines with the concurrent development of lymphocytic inflammation. Further, significant increases in both airway obstruction and airway hyper-reactivity correlate with toxin-induced inflammation. Inflammatory responses and changes in airway physiology elicited by rCARDS toxin are similar to those observed during *M. pneumoniae* infection suggesting that the CARDS toxin plays a major role in the pathogenesis of *M. pneumoniae*-mediated disease.

## Methods

### Bacteria

*M. pneumoniae* clinical isolate S1 was grown in SP4 liquid media in 175 cm tissue culture flasks and quantified as previously described [27]. Prior to infection of mice, suspensions containing mycoplasmas were passed through 28 gauge needles to disperse aggregates. These suspensions were used immediately to infect mice.

*Recombinant CARDS toxin*: rCARDS toxin was produced and purified to homogeneity as we have previously described [27]. Recombinant toxin preparations were maintained in carrier fluid consisting of 50 mM Tris pH 7.4, 5% glycerol, all certified to be low endotoxin. All toxin preparations were determined to be low in endotoxin by Limulus assay (Lonza) and bio-assay. Preparations of toxin used in this study contained less than 1 eu endotoxin/mg protein as determined in the Limulus assay. Bio-activity of the toxin was determined by the toxin's ability to induce vacuolization in HeLa cells as described [27].

### Animals

#### (i) Mice

Five to ten week old female BALB/cJ mice were purchased from Jackson Laboratory (Bar Harbor ME). Animals were kept in an ALAC approved facility in isolator cages with free access to food and water at all times. Depending on the experimental protocol, mice were anesthetized either by inhalation of Isoflurane or by intraperitoneal injection of 0.5 ml of Avertin (2,2,2-tribromoethanol; 20 mg/ml in PBS; Sigma, St. Louis, MO). Mice were then either infected intranasally (IN) with 10^7^ CFU of *M. pneumoniae* or treated IN with the indicated molar amount of rCARDS toxin or HI toxin. The latter was prepared by boiling toxin for one hr. Solutions of rCARDS toxin or suspensions of *M. pneumoniae* were delivered in a total volume of 30 µl applied in 15 µl/nare. Depending on experimental needs, mice were euthanized with either an overdose of Isoflurane (Iso-Thesia; Vetus Animal Health, Burns Veterinary Supply, Inc., Westbury, NY) or an intraperitoneal injection of 75 mg/kg ketamine and 5 mg/kg acepromazine followed by cardiac puncture at 0, 2, 4, 6, 14 and 37 days after toxin treatment. All experiments were performed in accordance with Institutional Biosafety Committee and Institutional Animal Use and Care Committee protocols established at The University of Texas Health Sciences Center at San Antonio and University of Texas Southwestern Medical School.

#### (ii) Baboons

Two adult female baboons (*Papio sp.*) were housed at the Southwest Foundation for Biomedical Research (SWFBR) in San Antonio, TX. Baboons were treated in accordance with guidelines established in the Weatherall report “The use of non-human primates in research”. Animals were group housed until used in experiments and had free access to food, water, and toys and were monitored during study by the veterinary staff. Baboons received ketamine sedation prior to handling for experimental procedures and were euthanized by phenobarbital overdose. All experiments were performed in accordance with Institutional Biosafety Committee and Institutional Animal Use and Care Committee protocols established at The Southwest Foundation for Biomedical Research.

### Cell culture

HeLa cells were cultured in DMEM medium supplemented with 10% heat inactivated fetal bovine serum. Cells were maintained in a 37°C humidified incubator with air and 5% CO_2_ and used at 60–75% confluence.

### Bronchoalveolar lavage (BAL)

#### (i) Mouse bronchoalveolar lavage fluid (BALF)

To obtain BALF, mice were euthanized, and a 1-cm longitudinal incision was made to expose the trachea. Bronchoalveolar lavage was performed by catheterizing the trachea using 18-gauge Angiocaths (Becton-Dickinson Infusion Therapy Systems, Inc., Sandy, UT) as we have described in detail before [30]. Each mouse was lavaged with a single pass of 1-ml of PBS with protease inhibitors (pepstatin, phenylmethylsulfonyl fluoride, aprotinin, and leupeptin [Sigma, St. Louis, MO]). BALF samples were placed immediately on ice, filtered with 0.2-µm syringe filters (SFCA;Fisher Scientific, Pittsburg, PA), and stored at −80°C for future analysis. Results reported here are representative of at least two independent experiments performed with 5–10 animals per treatment group and time.

#### (ii) Baboon BALF

Baboons were sedated prior to oral intubation and bronchoscopy using an Olympus bronchoscope. Baboons were lavaged with 20 ml of saline with a return of 10 ml of BALF. Following acquisition of baseline BALF specimens from the left lower lobe, one animal received 700 pmol of rCARDS toxin in carrier fluid in the right lower lobe (RLL) bronchus, and the control baboon received the same amount of HI rCARDS toxin in the RLL. Thereafter, BALF specimens were obtained on days one and two from both lower lobes and frozen at −80°C for future analysis.

### Cytokine analysis

#### (i) Luminex analysis

BALF samples from each treatment group were assayed to determine the presence of 21 different mouse cytokines and chemokines using a Bio-Rad mouse array for 21 analytes as recommended by the manufacturer. Baboon cytokine and chemokine levels were analyzed by Luminex assay for the expression of 21 different cytokines and chemokines using a validated primate panel developed at the Southwest Foundation for Biomedical Research (SWFBR) and performed by the Department of Immunology Core Facility at the SWFBR [31], [32], [33]. Five to ten mouse samples per treatment group per time point were assayed, and baboon samples were analyzed in duplicate from each lobe.

#### (ii) ELISA

**E**nzyme-linked immunosorbent assays (ELISA) was used to determine the concentration of select mouse cytokines. All other cytokine measurements were done by Luminex assay. Samples were diluted as appropriate and used in ELISA for TNF-α, ΚC, MCP-1, and IL-6, (R&D Systems, Minneapolis, MN or BD Pharmingen). BALF samples (5 to10) per time point were evaluated, and each sample was assayed in triplicate. Each experiment was done at least twice, and representative data are presented.

### Histopathology

Following intranasal instillation of rCARDS toxin (43.7, 175, or 700 pmol), mouse lungs were harvested at different times, and the gross appearance of each lobe was evaluated at necropsy. Tissue samples were obtained at 1, 4, 7, 14, and 37 days post inoculation. The right or left lung was fixed intrabronchially with 10% neutral buffered formalin solution. Tissue blocks were processed and embedded in paraffin, from which 4 um sections were cut and stained with hematoxylin and eosin. The histopathological findings and the grading of the lesions were done by a pathologist (JJC) who was blinded to the experimental treatment of the animals from which specimens were derived. Because the number of blocks was variable from each lung, the lesions were graded as normal, mild, moderate and severe. A severe designation was assigned when diffuse lesions of peribronchiolar and bronchial infiltrates and pneumonia were seen in over 50% of the lung lobe fragments; a moderate designation was used when focal lesions of peribronchiolar and bronchial infiltrates and/or pneumonia were present in 25 to 50% of the lung; and a mild designation was assigned to the presence of a single pneumonia site in one lobe piece or several localized lymphoid lesions in less than 25% of the lung. The counting technique used to grade cellular lesions was the method of Cimolai, et al [34], which was modified due to the variable lung surface available for analysis. Images were captured digitally with a Zeiss Axioscope 2 microscope equipped with a digital camera and processed using the Axiovision V.4 software suite (Carl Zeiss, Inc., Thornwood,NY).

### Airway function

Changes in mouse airway function after toxin exposure were determined by unrestrained whole-body non-sedated plethysmography as we have previously published [11], [12], [13], [14], [15], [16]. Animals were treated with various concentrations of toxin or carrier fluid, and baseline airway obstruction (AO) was determined by measuring the enhanced pause (P_enh_), a dimensionless value representing the ratio of peak expiratory flow to peak inspiratory flow and a function of the timing of expiration. P_enh_ correlates with pulmonary airway obstruction and hyperreactivity, which has been previously validated in animal models of airway hyperreactivity (AHR) [35], [36], [37], [38]. AHR was measured after aerosolized methacholine (100 mg/ml) challenge. Mice were serially analyzed for AO and AHR by plethysmography on days 2, 4, 7, 14, 21, 28 and 37 days.

### Statistical analysis

All results were expressed as the mean +/− standard deviation. Statistical differences were determined using a two-tailed Student *t-*test, a Mann-Whitney U test, or Kruskal-Wallis ANOVA on Ranks followed by Dunn's Method with GraphPad In-Stat3 (GraphPad Software). A *P* value of <0.05 was considered significant.

## Results

### CARDS toxin properties

**Figure 1:**
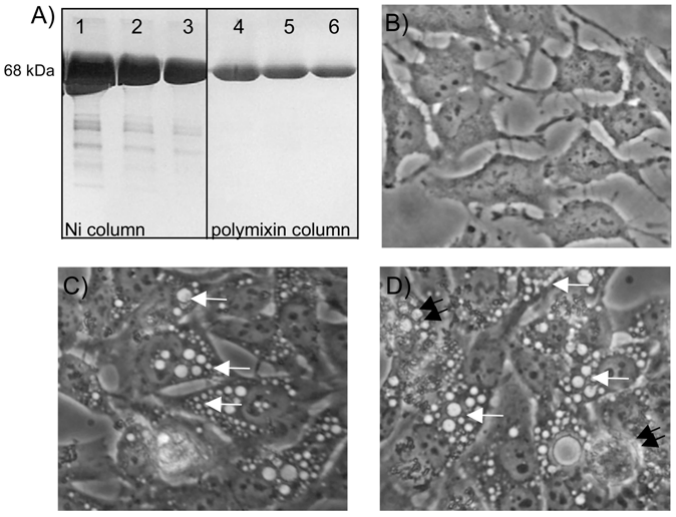
Vacuolation activity of rCARDS toxin. A) SDS-PAGE analysis of the peak fractions of rCARDS toxin after both nickel (Ni) affinity resin and polymixin columns reveals a single protein species of the appropriate size. B) HeLa cells treated with 700 pmol of HI toxin for 16 hrs serve as a negative control for assessment of vacuolization by microscopy. C and D) HeLa cells treated with 175 and 700 pmol of rCARDS for 16 hrs, respectively, exhibit a dose-dependent increase in numbers and sizes of vacuoles (small [175 pmol] and large [700 pmol] white arrows) relative to the negative control cells. Also, note the cytotoxic effect of rCARDS (double black arrows).

Previously, we reported that rCARDS toxin induces vacuoles in mammalian cell lines and ADP-ribosylates host target proteins [27]. To confirm the ability of each recombinant preparation of CARDS toxin to elicit responses in human cells, individual lots of toxin were tested for the ability to induce vacuoles in HeLa cells (Figure 1). Purified rCARDS toxin appears as a single species of 68 kDa when separated by SDS-PAGE and visualized by Coomassie blue staining confirming the purity of the toxin (Figure 1A). Passage of rCARDS toxin through the polymixin columns, which was used to reduce endotoxin levels, had no effect (Figure 1A). Since this toxin was used for in vivo studies, we measured contamination with endotoxin or other immune-stimulatory molecules by Limulus assay and bioassay. Both active and HI toxins have less than 1 eu of endotoxin/mg protein as determined by Limulus assay (not shown). HeLa cells treated with 175 or 700 pmol of active CARDS toxin for 16 hrs. develop vacuoles in a dose dependent manner in contrast to HI toxin used as a control in these studies (Figure 1B–D). Altogether these data suggest that the rCARDS toxin preparation used in these studies is biologically active based on the ability to produce vacuoles in epithelial cells. Further, Limulus assays indicate that this toxin has very low quantities of endotoxin.

### Pulmonary cytokine response to rCARDS toxin

**Figure 2:**
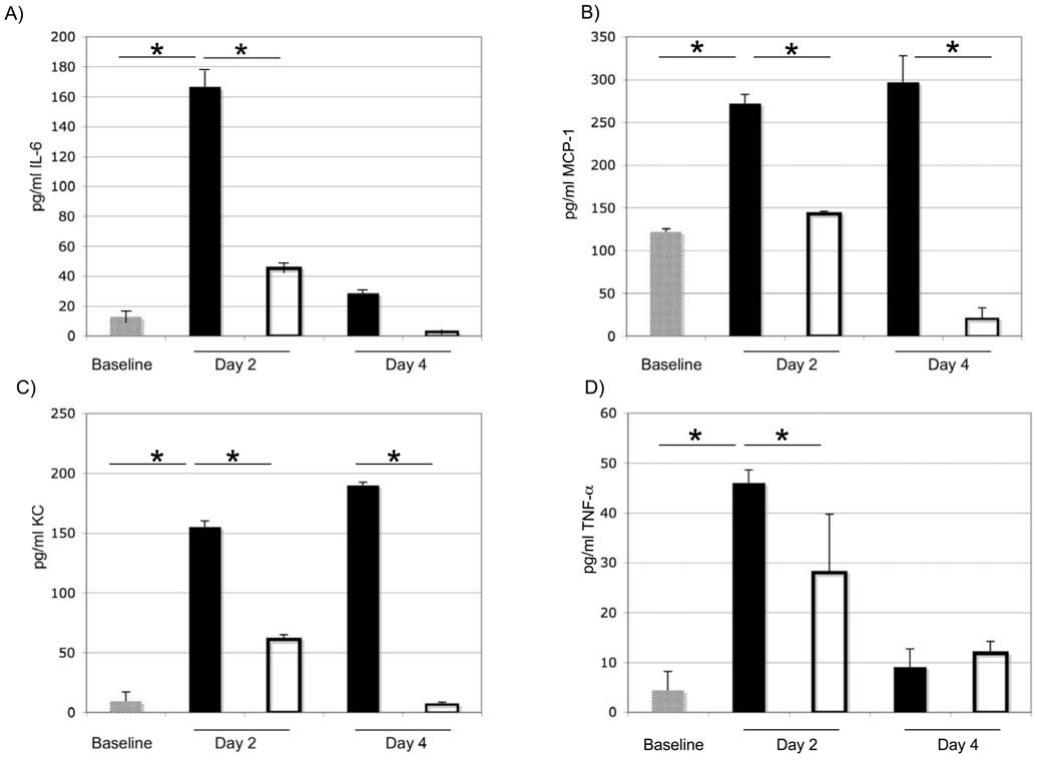
Changes in cytokine/chemokine concentrations in BALF after exposure to active CARDS toxin. Mice were treated IN with 700 pmol of rCARDS toxin (solid bars) or 700 pmol of HI toxin (open bars) and concentrations of cytokines/chemokines in the BALF were determined by ELISA. Baseline data are obtained from mice treated with saline for 2 days (stippled bars). A) Statistically significant (p<0.05) increase in IL-6 is observed relative to baseline and HI toxin that declines by day 4. B) Statistically significant (p<0.05) increase in MCP-1 is observed relative to baseline and HI toxin on days 2 and 4. C) Statistically significant (p<0.05) increase in KC is observed relative to baseline and HI toxin on days 2 and 4. D) Statistically significant (p<0.05) increase in TNF-α is observed relative to baseline and HI toxin that declines by day 4. Data are representative of two independent experiments with 5 to 10 mice/time point/treatment group. Data are presented as the average ± the standard deviation.

Using mouse models of *M. pneumoniae* pulmonary infection, we and others have demonstrated that the acute phase of infection is characterized by an increase in cytokine secretion into the BALF. In order to test the ability of rCARDS toxin to induce an immune response in the lung, we treated mice intranasally (IN) with a single dose of 175 or 700 pmol active rCARDS toxin, concentrations that elicited vacuolization (Figure 1). Control animals were treated with an equivalent dose of HI toxin. Overtly, mice exhibited no adverse response to toxin treatment; the animals remained active; the fur was not ruffled; and all animals survived until experimental end-points. Then, animals were euthanized and BALF collected for cytokine analysis on days 2, 4, 7, and 14-post toxin exposure. Cytokine concentrations in the BALF were determined using ELISA or Luminex assays as we have previously described [14], [15], [30], [39]. Specifically, ELISA was used to determine the concentrations of IL-6, TNF-α, MCP-1, and KC on days 0, 2, and 4 post-toxin treatment, and Luminex was used to determine the concentrations of IL-1α, IL-1β, IL-2, IL-3, IL-4, IL-5, IL-6, IL-9, IL-10, IL-12p40, IL-12p70, IL-13, IL-17, Eotaxin, G-CSF, GM-CSF, IFN-γ, KC, MIP-1α, MIP-1β, RANTES, TNF-α, and PDGF on days 0, 2, 4, 7, and 14. There were no significant differences in the concentrations of IL-2, IL-3, IL-4, IL-5, IL-9, IL-10, IL-13, Eotaxin, GM-CSF, IFN-γ, or PDGF when BALF from active toxin and HI control mice were compared (not shown). However, active toxin-treated mice showed a dose-dependent cytokine response, with the most robust responses observed in animals exposed to 700 pmol rCARDS when compared to animals treated with 175-pmol toxin. Cytokine responses peaked on day 2 post-exposure and declined thereafter with most cytokine levels returning to baseline by day 4 post-exposure. As shown in Figure 2 and Table 1, the response was predominantly a pro-inflammatory T-helper type 1 response with statistically significant (p<0.05) increases in the concentrations of IL-1α and β, IL-6, 12, 17, and TNF-α ranging from 2 to 166 fold over baseline. In addition to increases in pro-inflammatory cytokines, there were 6 to 84 fold increases in growth factors and chemokines in the BALF of mice treated with active rCARDS toxin including G-CSF, KC, MCP-1, MIP-1α, and MIP-1β (Figure 2 and Table 1). Both KC and MCP-1 had a more prolonged secretion into the BALF, being elevated until at least day 4 post-treatment (Figure 2B and C). Unlike most of the other cytokines that exhibited peak expression at day 2 post-treatment and then rapidly returned to baseline, 700 pmol CARDS toxin was able to induce a dose-dependent and sustained expression of IL-12p40 and KC that lasted until day 7 post-exposure (Figure 2 and Table 1). The patterns of cytokine expression detected by ELISA (Figure 2) and Luminex (Table 1) were in good agreement although the magnitude of the cytokine responses cannot be directly compared since the data are from separate experiments.

**Table 1:** Cytokine Levels in the Mouse Bronchial Alveolar Lavage Fluid after Exposure to CARDS Toxin.

700pmol CARDS									
Day		2	2	4	4	7	7	14	14
Cytokine^a^	Baseline^b^	Active	HI^c^	Active	HI	Active	HI	Active	HI
IL-1α	2±0.8	309±175*	29±49	10±6	7±2	7±1	4±0.4	0.8±0.7	2±1.4
IL-1β	13±0.2	123±69*	23±6	19±0.4	20±1	21±1	19±1	12±0.4	12±0.7
IL-6	3±0.8	49±44*	6±4	5±0.5	4±0.3	5±0.8	4±0.5	0.8±0.7	2.1±1.4
IL-12p40	6±0.5	105±41*	9±10	95±105*	5±2.5	17±9	7±6	6±3	5±1
IL-12p70	5±2	11±3*	3±3	2±1	2±1	2±1	2±1	5±1	5±1
IL-17	0.9±0.2	15±10**	5±8	7±8	2±2	2±2	1±1	0.6±0.3	0.5±0.5
G-CSF	6±0.1	509±444*	18±18	30±27	10±2	10±0.9	8.5±0.1	6±0.1	6±0.1
KC	6±0.2	59±33*	11±7	43±31*	9±2	42±40	12±7	8±3	9±4
MIP-1α	8±0.7	210±110*	15±26	17±15	7±0.4	10±11	4±3	8±3	8±1
MIP-1β	12±0.6	70±58	18±2	19±2	16±0.1	18±2	16±0.4	12±0.5	8±0.9
RANTES	4±0.1	66±30*	8±2	45±46	7±0.9	12±5	7±1	3±0.8	4±0.2
175pmol CARDS									
Day		2	2	4	4	7	7	14	14
Cytokine	Baseline	Active	HI	Active	HI	Active	HI	Active	HI
IL-1α	2±0.8	7±2	12±5	5±0.6	6±1	6±3	5±2	2±0.9	3±1.4
IL-1β	13±0.2	20±0.9	23±3	19±0.6	19±0.5	16±3	13±2	12±0.2	12±0.7
IL-6	3±0.8	5±0.4	4±0.3	5±0.5	4±0.3	6±2	4±0.5	2±1	3±2
IL-12p40	6±0.5	9±6	5±2	3±0.1	4±0.2	5±2	6±0.3	2±1	3±1
IL-12p70	5±2	3±0.7	2±1	2±1	2±2	4±2	5±1	4±1	7±4
IL-17	0.9±0.2	2±0.5	2±0.5	0.6±0.5	0.5±0.5	0.6±0.6	1±0.2	0.9±0.2	1±0.3
G-CSF	6±0.1	10±1	11±1	9±0.5	9±0.2	7±1	6±0.1	6±0.08	6±0.7
KC	6±0.2	9±1	10±3	8±0.5	7±0.2	10±2	6±0.5	8±2	6±0.7
MIP-1α	8±0.7	6±5	2±2	7±0	4±2	7±4	7±0.3	12±0.7	8±1
MIP-1β	12±0.6	17±0.4	16±0.1	16±0.1	16±0.1	15±2	12±0.7	12±0.7	12±0.5
RANTES	4±0.1	8±1	7±0.2	7±0.1	7±0.2	6±2	4±1	4±0.1	4±0.1

a Cytokines showing an increase over basal levels when animals are exposed to active CARDS toxin. Representative data from two independent assays with 5 to10 mice/time point/treatment group each are presented. Data are reported as pg/ml and presented as the average ± the standard deviation.

b Baseline measurements were obtained from 5 animals treated with saline for two days.

c HI toxin was boiled for one hour prior to use.

*p<0.05 comparing active toxin to baseline or a similar amount of HI toxin.

**p<0.05 comparing active toxin to baseline.

The pattern of cytokine responses and the ability of mice to respond to rCARDS toxin may not be reflective of the human response to toxin exposure. While it is not currently possible to directly test human responses to rCARDS toxin, we were able to test the response of non-human primates (baboons). Clinically, the baboons tolerated the procedures well and remained afebrile with normal chest radiographs (not shown). The concentration of cytokines in the BALF after treatment with active rCARDS or HI rCARDS toxin was determined by Luminex assay using a validated panel as described [31], [32], [33]. As shown in Table 2, elevations in cytokine and chemokine concentrations were noted in the active rCARDS toxin-treated animal at days 1 and 2, but not in the control: G-CSF (40 fold), IL1Ra (10 fold), IL6 and IL8 (333 and 100 fold, respectively), MIP-1α (5 fold), and RANTES (9 fold) (Table 2). Overall, the responses in the baboon closely mimicked those observed in the mouse suggesting a conserved mechanism of immune activation by the CARDS toxin.

**Table 2:** Cytokine Levels in the Baboon Bronchial Alveolar Lavage Fluid after Exposure to CARDS Toxin.

Cytokine^a^	Baseline^b^	Baseline HI^c^	Day 1^d^	Day1 HI^d^	Day 2^d^	Day 2 HI^d^
IL-12p40	7	7	3	1.28	3	1
IL-17	10	10	7.4	10	2.5	10
IL-1ra	95	61	9.8	7	4	0.93
IL-6	6	6	333.6	14.7	320	1
IL-8	6	6	97.6	23	48	1
IFN-γ	6	6	13	6	1.5	6
MCP-1	453	619	1.3	1.6	1.3	0.5
MIP-1α	69	6	4.8	23	3	2
RANTES	13	11	8.5	1.4	3.1	0.36
G-CSF	17	19	40.8	2.3	8.0	0.68

a Cytokines expressed at days 1 and 2 after treatment with 700 pmol active rCARDS toxin or 700 pmol heat inactivated toxin.

b Baseline values obtained in the pre-procedure BALF expressed as pg/ml.

c Baseline values obtained in the pre-procedure BALF for HI toxin expressed as pg/ml.

d Cytokine levels were determined by Luminex assay and are presented as fold increases over baseline. No changes were detected in: M-CSF, IFN-α, IL-1β, IL-2, IL-4, IL-5, IL-10, IL-18, MIP-1β, TNF-α, TNF-β.

### rCARDS toxin induces lymphocytic inflammation

**Figure 3:**
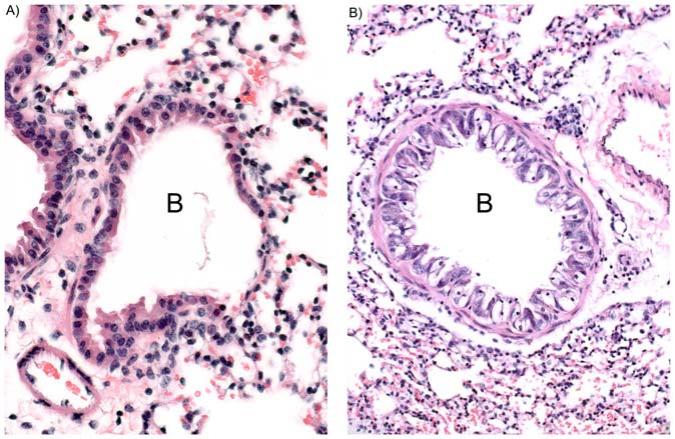
Vacuolization of bronchiolar epithelium after CARDS toxin exposure. Two days after IN exposure to 43.7 pmol of active rCARDS toxin or HI toxin, histological changes in lung specimens were evaluated. A) Bronchiolar epithelial cells appear normal in the HI toxin–treated lung. B) Extensive vacuolization of the airway epithelium is evident in rCARDS toxin-treated lung. B = bronchiole. Hematoxylin and eosin, X 40 & 20 original magnification, respectively.

Infection of mice with *M. pneumoniae* leads to dynamic inflammatory changes in pulmonary histopathology [34]. Based on observations with *M. pneumoniae* infections, it was important to examine the histopathology that follows CARDS toxin exposure. We treated mice IN once with 175 or 700 pmol active rCARDS or HI toxin. On days 2, 4, 7, 14, and 37 post toxin, mice were euthanized and the lungs prepared for routine hematoxylin and eosin staining by fixation in 10% neutral buffered formalin (NBF) prior to being embedded in paraffin, sectioned, and stained. No histologic abnormalities were present in saline treated control lungs, and heat-inactivated toxin treated lungs showed minimal numbers of lymphocytes in peribronchiolar and perivascular sites (Figure 3A and not shown).

**Figure 4:**
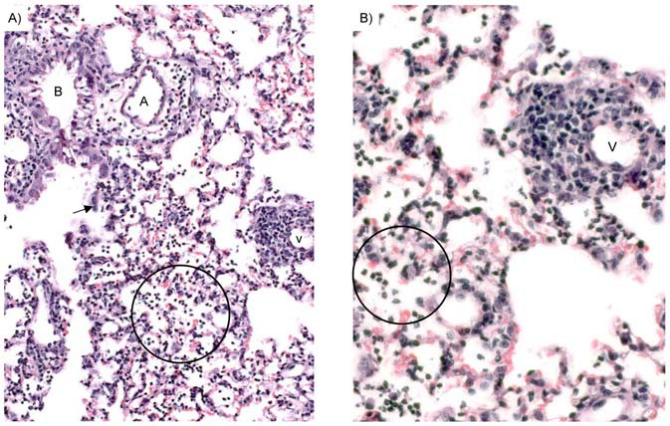
Histological changes in lungs after exposure of mice to 700pmol of rCARDS toxin for two days. A) Epithelial vacuolization in the terminal bronchiole (B), ulceration of the epithelium, and neutrophilic exudates are evident at the respiratory bronchiolar level (arrow). The surrounding alveolar spaces contain exudates of neutrophils and rare alveolar macrophages (circles). Whereas the pulmonary artery (A) is enclosed by edema and scattered inflammatory cells, the pulmonary venule (v) shows a mixed perivascular infiltrate of mononuclear and a few acute inflammatory cells. B) is a 4-fold magnification of the area enclosed within the circle indicated on Panel A. Hematoxylin and eosin, X 10 & 40 original magnification, respectively.

Dramatically, lungs from mice treated with 43.7 pmol, 175 pmol or 700 pmol CARDS TX showed striking vacuolization of bronchial and bronchiolar epithelium at the 2-day study period which disappeared in all study groups at 4 days (Figures 3B and 4A). This finding mimicked previously reported changes in the epithelium of baboon tracheal rings treated with CARDS toxin [27] but is the first demonstration of the ability of rCARDS toxin to induce vacuolization in vivo. In the lungs that received 175 pmol CARDS TX, scattered lymphocytes, but no neutrophils, were evident in the septae and occasional airways and vessels at days 2 and 4 (not shown). At day 7, patchy, small numbers of lymphocytes were present, but at 14 days the lungs appeared near normal (not shown).

**Figure 5:**
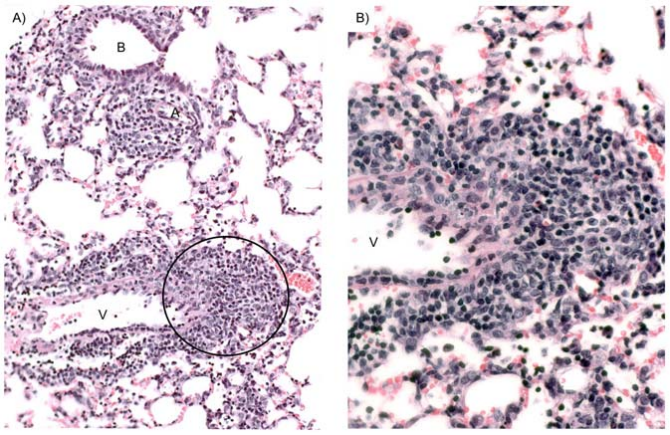
Lymphocytic inflammation four days after treatment with 700 pmol of rCARDS toxin. A) Focal collections of neutrophils in alveolar spaces are evident. The epithelium of the bronchiole lacks the vacuolization lesion, and the pulmonary artery (A) and vein (V) show multilayered mononuclear cells (circle) with predominantly small lymphocytes and rare neutrophils. B) is a 2-fold magnification of Panel A. Hematoxylin and eosin, X 20 & 40 original magnification, respectively.

**Figure 6:**
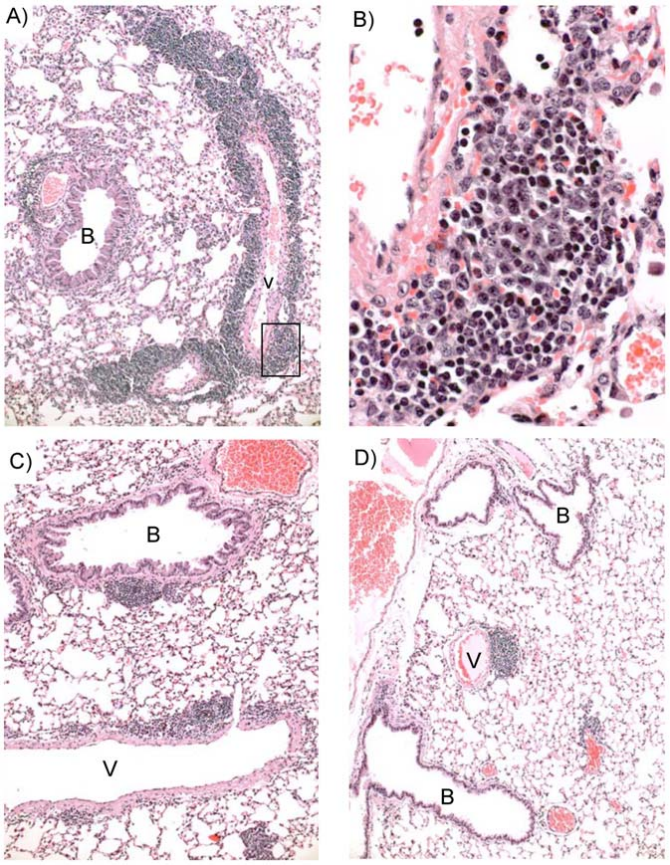
Peak inflammatory lesions and resolution of cellular infiltration 7 to 37 days after treatment with 700 pmol rCARDS toxin. A) 7 days post-toxin treatment, focal pneumonitic exudates are evident in alveoli, but the striking lesions at this time point are the condensed perivascular and fewer peribronchiolar lymphocytic infiltrates. B) The rectangular area in Panel A is a collection of lymphoblasts present amongst the small lymphocytes (magnified 6-fold). C) 14 days post-treatment, the perivascular and peribronchial/iolar lymphoid infiltrates are much fewer in distribution and number. D) 37 days post-toxin treatment perivascular and peribronchial lesions are rare but still present in rCARDS toxin-treated lungs. artery = A, B = bronchiole, V = vein. Hematoxylin and eosin, X 10, 60, 10, and 10 original magnification, respectively.

**Figure 7:**
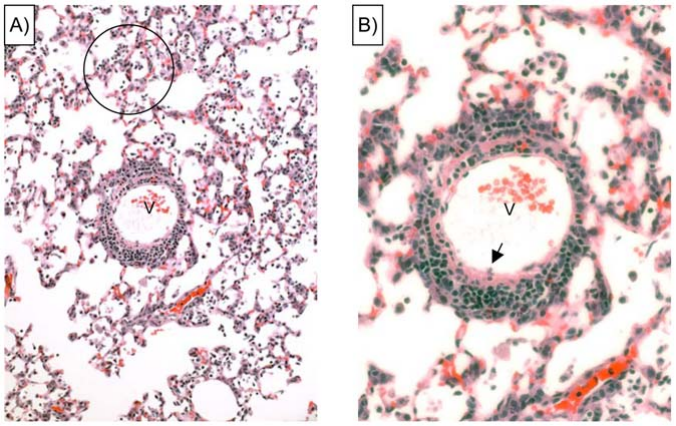
Mouse perivascular lesions evident in the lung 4 days after *M. pneumoniae* infection. A) Low power micrograph shows scattered alveolar exudates (circle) proximal to a venule (v) with a mural cellular infiltrate of small mononuclear cells, predominately small lymphocytes. B) is a 4-fold magnification of Panel A) showing that the vessel wall is infiltrated with multilayered small lymphocytes. The endothelium is edematous, and adherent inflammatory cells are occasionally evident (arrow). Compare this histopathology to similar lesions induced by active rCARDS toxin presented in Figure 5A & B. Hematoxylin and eosin, original magnifications 10× and 40× respectively.

**Figure 8:**
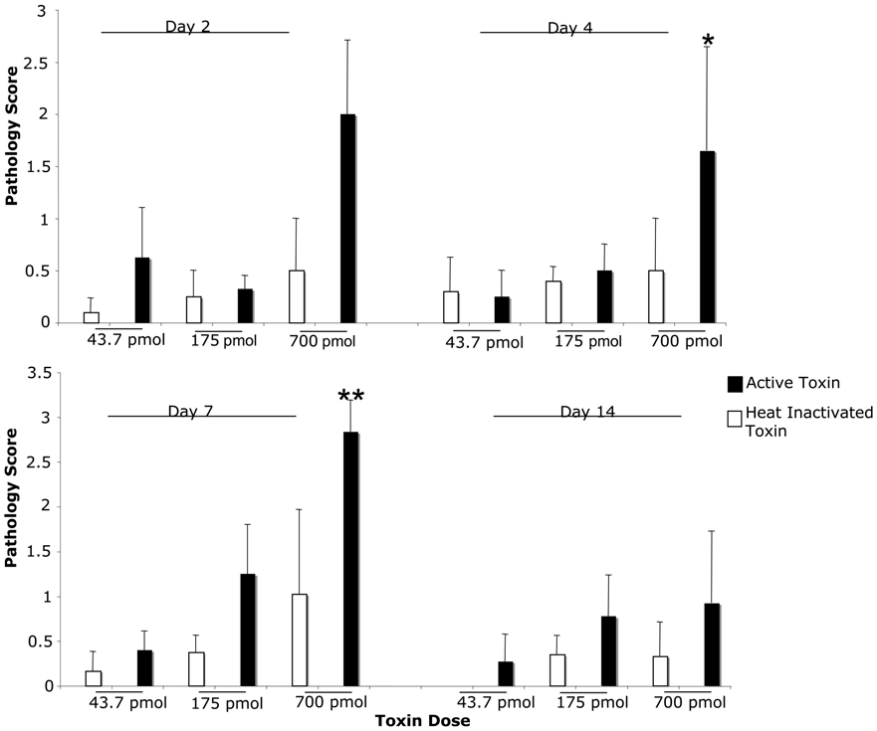
Quantifiable inflammatory histopathology observed in rCARDS toxin-treated mice. Histopathology was scored in a blinded fashion, and sections were given a numerical score as described in the Materials and Methods. The most significant changes were observed in animals treated with 700 pmol of rCARDS on days 4 and 7 post-treatment. Data represents the average ± standard deviation with 8 to10 mice/time point/treatment group. Data sets were analyzed for statistical significance by comparing animals treated with the indicated amount of toxin to the corresponding group of animals treated with HI toxin * = p<0.05, ** = p<0.005.

The histopathological findings in the lungs treated with 700 pmol CARDS TX showed striking histopathological lesions that were very similar to those we have reported in lungs inoculated with live *M. pneumoniae* organisms. At 2 days post-inoculation with 700 pmol CARDS toxin, an intense inflammatory response was evident focally in the walls of pulmonary vessels and airways, alveolar spaces, and the interstitium (Figure 4A). The dominant cell type was the neutrophil, but many small lymphocytes were also present, especially around some vessels, probably venous, with fewer in the edematous walls around airways and the accompanying pulmonary artery branch. The pneumonitic exudates in alveolar spaces contained numerous neutrophils (Figure 4A and B circles). As noted above, the airways were lined by injured respiratory epithelium that showed vacuolization, cytoplasmic degenerative changes and cilial damage. At day 4, the striking lesion was the multilayered collections of small lymphocytes that encircled the pulmonary arteries and veins with small peribronchial/iolar accumulations (Figures 5, 6, and 7). Foci of pneumonia were present, but the respiratory epithelium lacked the severe vacuolization detected at day 2. The pronounced perivascular infiltrates present four days after exposure to 700 pmol rCARDS are similar to lesions observed during infection. For example, lymphocytes are readily observed both perivascularly and tightly attached to the endothelium adjacent to the infiltrates (Figure 7A and B, arrows). At day 7, the lymphocytic infiltrates were more extensively layered around vessels and bronchioles (Figure 6A and B). Although a few lymphocytes had appeared more blastic at day 4, this change to larger cell size, more abundant cytoplasm and enlarged nuclei with prominent nucleoli was particularly striking at day 7. Foci of these reactive lymphocytes were embedded in the perivascular infiltrates of small lymphocytes (Figure 8B). At day 14, the perivascular lymphoid aggregates were waning in number but still numerous around vessels and occasional airways (Figure 6C). At day 37, only scattered aggregates of perivascular lymphocytes were evident (Figure 6D).

On days 4 and 7 post-treatment, quantitative scoring of the lesions revealed statistically significant differences in the histological scores of the animals treated with 700 pmol of rCARDS toxin relative to the animals treated with an equivalent amount of heat-inactivated toxin (Figure 8).

**Figure 9:**
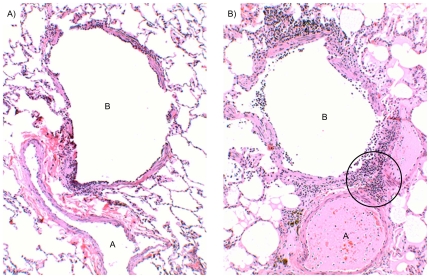
rCARDS toxin induction of inflammatory changes in primate lungs. 700 pmol of active or HI CARDS toxin were introduced directly into the baboon lung. A) Normal appearing bronchioles (B) are evident in the HI toxin treated lung. B) Intrabronchial instillation of CARDS toxin results in a lymphocytic bronchiolitis and an early perivascular component (circle). artery = A. Hematoxylin and eosin, X10 original magnification, respectively.

The lung of the mouse is anatomically different from that of humans and other primates raising the possibility that the primate lung may respond differently to rCARDS toxin exposure. To investigate the pathological changes in primates, sections of baboon trachea, right and left lower lobe bronchi, and distal bronchi and bronchioles were obtained (Figure 9). Histopathologically, trachea and bronchi of the HI control showed focal epithelial loss and scattered eosinophils secondary to the aggressive lavage procedures (not shown). Distal bronchi and bronchioles showed intact epithelium and no mural inflammation (Figure 9A), with only scattered small collections of bronchus-associated lymphoid tissue in some bronchiolar walls. In active toxin-treated baboon specimens, the trachea, right and left main bronchi and more distal bronchi showed extensive denudation of epithelium and increased numbers of lymphocytes and scattered eosinophils in the submucosa (not shown). A lymphocytic bronchiolitis was identified in numerous bronchioles, and foci of alveolar edema were present (Figure 9B). These data suggest that the CARDS toxin is capable of eliciting a response in the baboon that is consistent with what is observed in human *M. pneumoniae* infection and in mouse models of both infection and intoxication.

### Toxin mediated changes in airway function

The primary function of the lung as an organ of gas exchange can be significantly impacted by both acute and chronic inflammation. The robust changes in cytokine expression and histopathology after rCARDS toxin treatment suggest that CARDS toxin has the potential to induce significant immune pathology in the lung. Changes in airway function can be a direct result of inflammation, as is the case with the asthmatic lung, and may manifest as AO and AHR. Therefore, we monitored changes in airway function using unrestrained whole-body non-sedated plethysmography as we and others have described previously [11], [13], [14], [15], [16], [35], [36], [37], [38]. We chose this method because it allows us to serially monitor the same animals over time, thus giving us the advantage of following disease development and resolution in the same cohorts of animals. Further, a rigorous direct comparison of Penh versus invasive measurement of lung resistance in mice was published (39). This direct comparison found that for BALB/c mice the correlation coefficient was 0.809 between Penh and invasive measurement of lung resistance (*P*<0.05).

**Figure 10:**
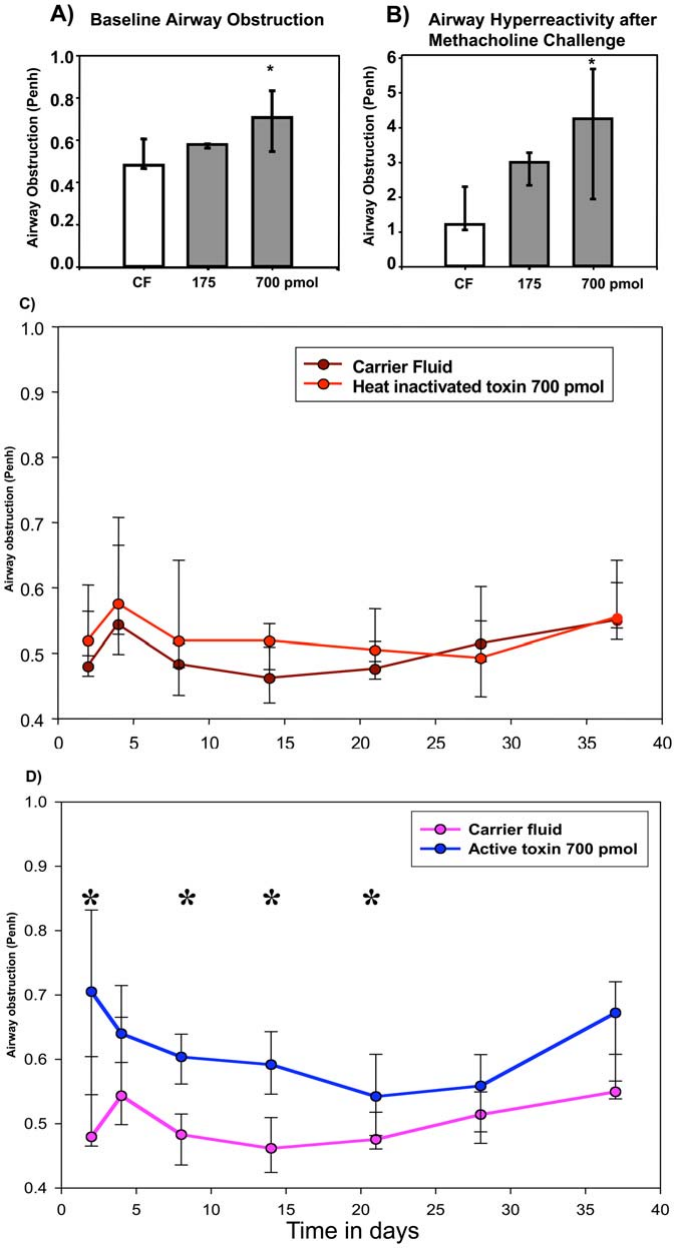
Airway obstruction and airway hyperreactivity in mice after pulmonary exposure to rCARDS toxin. A) Baseline AO is presented in response to different amounts of rCARDS toxin 2 days post-treatment (* = p<0.05) relative to carrier fluid (CF). B) Methacholine responsive AHR is observed 2 days after treatment with the indicated amount of rCARDS toxin (* = p<0.05) relative to CF. C) Lack of AO is observed in response to 700 pmol of HI toxin or CF. D) AO remains detectable over a 37 day time course with mice treated with 700 pmol of rCARDS or CF. * = p<0.05. Data are presented as the average ± standard deviation with 5 to 10 BALB/c mice/time point/treatment group.

BalbC/J mice were treated IN with 175 or 700 pmol rCARDS toxin or carrier fluid, and then baseline AO and AHR were determined on day 2. As shown in Figures 10A and B, 700 pmol rCARDS toxin induced statistically significant (p<0.05) changes in both AO and AHR two days after toxin exposure. There was no difference in AO between animals treated with HI CARDS toxin or carrier fluid (Figure 10C). Therefore, carrier fluid was used in all subsequent studies. To determine the kinetics of the changes in airway function, mice were treated with a single dose of 700-pmol rCARDS toxin and then AO was monitored 2, 4, 7, 14, 21, 28, and 37 days post treatment. rCARDS toxin induced a prolonged statistically significant (p<0.05) change in AO that lasted 21 days post-toxin treatment (Figure 10D). Altogether, these data indicate that a single application of active rCARDS toxin is capable of inducing prolonged AO and transient AHR.

## Discussion

This study represents the first analysis of the interaction of *M. pneumoniae* CARDS toxin with the immune system of an experimental animal model. While many bacterial pathogens produce toxins that modify the host response to infection, the recently discovered CARDS toxin, in contrast to many bacterial toxins, is unique in that it is both a vacuolating and ADP-ribosylating toxin. Currently, it is not clear how ADP-ribosylation or the phenotype of vacuolation correlates with the ability of the CARDS toxin to induce inflammation. However, the data presented in this study indicate that CARDS toxin is capable of inducing pro-inflammatory cytokine expression in the lungs of mice and baboons. In mice treated with a single dose of rCARDS toxin, the toxin induces a transient pro-inflammatory response characterized by increased expression of IL-1, 6, 12p40, 17, and TNF-α as well as a number of inflammatory chemokines. In the baboon, G-CSF, IL-1ra, 6, 8, MIP-1α, RANTES, and IFN-γ were readily detectable after rCARDS toxin treatment. The pattern of pro-inflammatory cytokine expression in both mice and baboons is remarkably similar to that observed during human pulmonary infection with *M. pneumoniae* [3], including the prolonged expression of IL-12p40 after rCARDS toxin exposure. The relatively high levels of IL-12p40 but lower levels of IL-12p70 may suggest a role for IL-23, but this remains to be investigated. Hardy and co-workers have demonstrated that mice lacking IL-12 infected with *M. pneumoniae* have less severe disease and rapidly resolve inflammatory changes in lung histology [15]. In contrast, treatment of mice with rIL-12 during the acute phase of infection prolongs acute disease, increases pro-inflammatory cytokine levels, and exacerbates inflammatory pathology as well as changes in airway function [14]. rCARDS toxin appears to be capable of inducing several but not all of the cytokines reported to be produced after *M. pneumoniae* infection, which is predictable given the complexity of mycoplasma antigens presented to the immune system during infection.

Clearly, exposure to rCARDS toxin not only increases pro-inflammatory cytokine levels but also leads to profound changes in inflammatory histopathology. In vitro the CARDS toxin is an ADP-ribosylating and vacuolating toxin that is capable of inducing vacuoles in both cell and organ cultures (27). Here we demonstrated that early after a single exposure to rCARDS toxin, frequent and consistent vacuolization of the bronchiolar epithelium in vivo occurs that resolves by day 4, which corroborates our initial studies in tissue and organ culture [27]. Interestingly, the kinetics of the resolution of the vacuolization is consistent with rapid regeneration of bronchiolar epithelium.

The CARDS toxin-mediated inflammatory infiltrates are very similar to those observed during *M. pneumoniae* infection, and the perivascular lesions suggest a hematological origin (Figure 7). rCARDS toxin eventually induces a robust peribronchial inflammation evident starting at day 4 post exposure. How specific cytokines and chemokines actually contribute to the development of these inflammatory infiltrates remains to be identified, but previous studies suggest that IL-12 might play a significant role [14], [15]. The cellular infiltrates are unusual in that they are predominantly lymphocytic in nature and increase in severity over a seven-day period in a dose-dependent manner following a single exposure to toxin. The presence of lymphoblasts after toxin exposure is uncommon during the host response to most bacterial toxins, and it is unclear what the significance of this finding is. However, it indicates a complex and interdependent relationship between *M. pneumoniae* CARDS toxin and the immune system. For example, the same lymphocytic aspect of the histopathology is observed following both *M. pneumoniae* infection and intoxication, suggesting a role for toxin during infection.

Pathophysiologically, exposure to CARDS toxin and the associated inflammatory responses in the lungs could account for the changes observed in pulmonary function after infection. Mouse models of chronic respiratory infection with *M. pneumoniae* have been established and have demonstrated that these chronically infected animals develop alteration in lung compliance, functional AO and AHR, as well as histological evidence for airway inflammation accompanied by fibrosis [10], [11], [12], [40]. Here we report that mice exposed to a single dose of rCARDS toxin develop statistically significant prolonged AO over 21 days and AHR at day 2-post exposure. The fact that AHR waned prior to resolution of AO indicates that AHR requires a sustained exposure to toxin, which would be typical of the infectious process. Prolonged AHR similar to what is observed during infection may also have been observed with a continuous toxin challenge. These data suggest that a single exposure to CARDS toxin is sufficient to cause significant changes in histopathology and airway function in mice that may be clinically relevant to a range of *M. pneumoniae*-associated sequelae.

*Mycoplasma pneumoniae* has been implicated as a causative agent or an exacerbating factor in a number of acute and chronic airway-associated inflammatory conditions ranging from tracheobronchitis and community acquired pneumonia to asthma and COPD in both children and adults [3], [5], [6], [7], [8], [41], [42]. In adults, *M. pneumoniae* has been detected in the airways of chronic, stable asthmatics using PCR with significantly greater frequency than in non-asthmatic control subjects [6], [43]. In a randomized, double blind, placebo-controlled trial of prolonged (6 week) clarithromycin therapy in 55 adult subjects with chronic, stable asthma [44] *M. pneumoniae* was detected by PCR in the airways of 23 of the 55 asthmatics. Prolonged clarithromycin therapy resulted in a significant improvement in pulmonary function (FEV_1_) only in the PCR-positive asthmatics (p = 0.05) and was without effect in the PCR-negative asthmatics (p = 0.85), directly linking *M. pneumoniae* and mycoplasma products to airway dysfunction. A conclusive causal link between *M. pneumoniae* infection and reactive airway disease is lacking but based on the ability of rCARDS toxin to induce robust inflammation and both AO and AHR in mice, it would be reasonable to predict that CARDS toxin would elicit similar responses in humans. Further, the improvement in airway function after antibiotic treatment (42) suggests that a virulence factor sensitive to protein synthesis inhibitors might play a role in the pathogenesis of disease. CARDS toxin is a likely candidate based on the in vivo effects of rCARDS toxin reported in this study and the fact that the magnitude of CARDS toxin expression by *M. pneumoniae* is markedly increased in vivo using the mouse model of infection relative to toxin expression during broth culture (T.R. Kannan et al Submitted).

Currently, there is no reliable way to estimate the total amount of CARDS toxin produced in vivo during *M. pneumoniae* infection. Therefore, it is difficult to make direct comparisons between treatment with rCARDS toxin and toxin produced during infection although the host response to rCARDS exposure or infection shares similarities (3,27). During infection many factors can lead to an inflammatory response, and it is unlikely that a single *M. pneumoniae* product accounts for the overall cytokine responses, the corresponding lymphocytic inflammation, as well as changes in airway function. During infection, the airway-associated histopathology and airway dysfunction, which we observed after rCARDS treatment alone, can be further amplified by other immuno-reactive mycoplasma products present at the site of infection. However, the results of this study suggest that the CARDS toxin is sufficient to induce major inflammatory phenotypes associated with *M. pneumoniae* infection in rodents and primates. These data support the concept that the CARDS toxin plays a fundamentally important role in the pathogenesis of *M. pneumoniae* infection and the sequelae associated with infection. Clinically, it is likely that CARDS toxin is a significant cause of human morbidity associated with *M. pneumoniae* infection.

## References

[1] Couch RB, Cate TR, Chanock RM (1964). Infection with artificially propagated Eaton agent (*Mycoplasma pneumoniae*). Implications for development of attenuated vaccine for cold agglutinin-positive pneumonia.. JAMA.

[2] Feizi T, Taylor-Robinson D (1967). Cold agglutinin anti-I and *Mycoplasma pneumoniae*.. Immunology.

[3] Waites KB, Talkington DF (2004). *Mycoplasma pneumoniae* and its role as a human pathogen.. Clin Microbiol Rev.

[4] Talkington DF, Shott S, Fallon MT, Schwartz SB, Thacker WL (2004). Analysis of eight commercial enzyme immunoassay tests for detection of antibodies to *Mycoplasma pneumoniae* in human serum.. Clin Diagn Lab Immunol.

[5] Johnston SL, Martin RJ (2005). *Chlamydophila pneumoniae* and *Mycoplasma pneumoniae*: a Role in asthma pathogenesis?. Am J Respir Crit Care Med.

[6] Martin RJ, Kraft M, Chu HW, Berns EA, Cassell GH (2001). A link between chronic asthma and chronic infection.. J Allergy Clin Immunol.

[7] Sabato AR, Martin AJ, Marmion BP, Kok TW, Cooper DM (1984). *Mycoplasma pneumoniae*: acute illness, antibiotics, and subsequent pulmonary function.. Arch Dis Child.

[8] Seggev JS, Lis I, Siman-Tov R, Gutman R, Abu-Samara H (1986). *Mycoplasma pneumoniae* is a frequent cause of exacerbation of bronchial asthma in adults.. Ann Allergy.

[9] Chu HW, Honour JM, Rawlinson CA, Harbeck RJ, Martin RJ (2003). Hygiene hypothesis of asthma: a murine asthma model with *Mycoplasma pneumoniae* infection.. Chest.

[10] Chu HW, Rino JG, Wexler RB, Campbell K, Harbeck RJ (2005). *Mycoplasma pneumoniae* infection increases airway collagen deposition in a murine model of allergic airway inflammation.. Am J Physiol Lung Cell Mol Physiol.

[11] Hardy RD, Jafri HS, Olsen K, Hatfield J, Iglehart J (2002). *Mycoplasma pneumoniae* induces chronic respiratory infection, airway hyperreactivity, and pulmonary inflammation: a murine model of infection-associated chronic reactive airway disease.. Infect Immun.

[12] Hardy RD, Jafri HS, Olsen K, Wordemann M, Hatfield J (2001). Elevated cytokine and chemokine levels and prolonged pulmonary airflow resistance in a murine *Mycoplasma pneumoniae* pneumonia model: a microbiologic, histologic, immunologic, and respiratory plethysmographic profile.. Infect Immun.

[13] Fonseca-Aten M, Rios AM, Mejias A, Chavez-Bueno S, Katz K (2005). *Mycoplasma pneumoniae* induces host-dependent pulmonary inflammation and airway obstruction in mice.. Am J Respir Cell Mol Biol.

[14] Salvatore CM, Fonseca-Aten M, Katz-Gaynor K, Gomez AM, Hardy RD (2008). Intranasal interleukin-12 therapy inhibits *Mycoplasma pneumoniae* clearance and sustains airway obstruction in murine pneumonia.. Infect Immun.

[15] Salvatore CM, Fonseca-Aten M, Katz-Gaynor K, Gomez AM, Mejias A (2007). Respiratory tract infection with *Mycoplasma pneumoniae* in interleukin-12 knockout mice results in improved bacterial clearance and reduced pulmonary inflammation.. Infect Immun.

[16] Woolard MD, Hardy RD, Simecka JW (2004). IL-4-independent pathways exacerbate methacholine-induced airway hyperreactivity during mycoplasma respiratory disease.. J Allergy Clin Immunol.

[17] Chu HW, Jeyaseelan S, Rino JG, Voelker DR, Wexler RB (2005). TLR2 signaling is critical for *Mycoplasma pneumoniae*-induced airway mucin expression.. J Immunol.

[18] Galanos C, Gumenscheimer M, Muhlradt P, Jirillo E, Freudenberg M (2000). MALP-2, a *Mycoplasma* lipopeptide with classical endotoxic properties: end of an era of LPS monopoly?. J Endotoxin Res.

[19] Into T, Nodasaka Y, Hasebe A, Okuzawa T, Nakamura J (2002). Mycoplasmal lipoproteins induce toll-like receptor 2- and caspases-mediated cell death in lymphocytes and monocytes.. Microbiol Immunol.

[20] Kraft M, Adler KB, Ingram JL, Crews AL, Atkinson TP (2008). *Mycoplasma pneumoniae* induces airway epithelial cell expression of MUC5AC in asthma.. Eur Respir J.

[21] Takeuchi O, Kaufmann A, Grote K, Kawai T, Hoshino K (2000). Cutting edge: preferentially the R-stereoisomer of the mycoplasmal lipopeptide macrophage-activating lipopeptide-2 activates immune cells through a toll-like receptor 2- and MyD88-dependent signaling pathway.. J Immunol.

[22] Wu Q, Martin RJ, Rino JG, Jeyaseelan S, Breed R (2007). A deficient TLR2 signaling promotes airway mucin production in *Mycoplasma pneumoniae*-infected allergic mice.. Am J Physiol Lung Cell Mol Physiol.

[23] Baseman JB, Reddy SP, Dallo SF (1996). Interplay between *Mycoplasma* surface proteins, airway cells, and the protean manifestations of *Mycoplasma*-mediated human infections.. Am J Respir Crit Care Med.

[24] Baseman JB (1993). The cytadhesins of *Mycoplasma pneumoniae* and *M. genitalium*.. Subcell Biochem.

[25] Baseman JB, Tully JG (1997). *Mycoplasmas*: sophisticated, reemerging, and burdened by their notoriety.. Emerg Infect Dis.

[26] Balasubramanian S, Kannan TR, Baseman JB (2008). The surface-exposed carboxyl region of *Mycoplasma pneumoniae* elongation factor Tu interacts with fibronectin.. Infect Immun.

[27] Kannan TR, Baseman JB (2006). ADP-ribosylating and vacuolating cytotoxin of *Mycoplasma pneumoniae* represents unique virulence determinant among bacterial pathogens.. Proc Natl Acad Sci U S A.

[28] Kannan TR, Provenzano D, Wright JR, Baseman JB (2005). Identification and characterization of human surfactant protein A binding protein of *Mycoplasma pneumoniae*.. Infect Immun.

[29] Krueger KM, Barbieri JT (1995). The family of bacterial ADP-ribosylating exotoxins.. Clin Microbiol Rev.

[30] Bubeck SS, Cantwell AM, Dube PH (2007). Delayed inflammatory response to primary pneumonic plague occurs in both outbred and inbred mice.. Infect Immun.

[31] Giavedoni LD (2005). Simultaneous detection of multiple cytokines and chemokines from nonhuman primates using luminex technology.. J Immunol Methods.

[32] McCurnin D, Clyman RI (2008). Effects of a patent ductus arteriosus on postprandial mesenteric perfusion in premature baboons.. Pediatrics.

[33] Thomson MA, Yoder BA, Winter VT, Giavedoni L, Chang LY (2006). Delayed extubation to nasal continuous positive airway pressure in the immature baboon model of bronchopulmonary dysplasia: lung clinical and pathological findings.. Pediatrics.

[34] Cimolai N, Taylor GP, Mah D, Morrison BJ (1992). Definition and application of a histopathological scoring scheme for an animal model of acute *Mycoplasma pneumoniae* pulmonary infection.. Microbiol Immunol.

[35] Gonzalo JA, Lloyd CM, Wen D, Albar JP, Wells TN (1998). The coordinated action of CC chemokines in the lung orchestrates allergic inflammation and airway hyperresponsiveness.. J Exp Med.

[36] Hamelmann E, Schwarze J, Takeda K, Oshiba A, Larsen GL (1997). Noninvasive measurement of airway responsiveness in allergic mice using barometric plethysmography.. Am J Respir Crit Care Med.

[37] Schwarze J, Hamelmann E, Bradley KL, Takeda K, Gelfand EW (1997). Respiratory syncytial virus infection results in airway hyperresponsiveness and enhanced airway sensitization to allergen.. J Clin Invest.

[38] van Schaik SM, Enhorning G, Vargas I, Welliver RC (1998). Respiratory syncytial virus affects pulmonary function in BALB/c mice.. J Infect Dis.

[39] Dube PH, Handley SA, Lewis J, Miller VL (2004). Protective role of interleukin-6 during *Yersinia enterocolitica* infection is mediated through the modulation of inflammatory cytokines.. Infect Immun.

[40] Liu J, Peng D, Zhu Z, Che D, Yang M (1998). The expression of PDGF-B chain mRNA in lung tissue from rats repeatedly infected with *Mycoplasma pneumoniae*.. J Tongji Med Univ.

[41] Lieberman D, Lieberman D, Printz S, Ben-Yaakov M, Lazarovich Z (2003). Atypical pathogen infection in adults with acute exacerbation of bronchial asthma.. Am J Respir Crit Care Med.

[42] Waites KB (2003). New concepts of *Mycoplasma pneumoniae* infections in children.. Pediatr Pulmonol.

[43] Kraft M, Cassell GH, Henson JE, Watson H, Williamson J (1998). Detection of *Mycoplasma pneumoniae* in the airways of adults with chronic asthma.. Am J Respir Crit Care Med.

[44] Kraft M, Cassell GH, Pak J, Martin RJ (2002). *Mycoplasma pneumoniae* and *Chlamydia pneumoniae* in asthma: effect of clarithromycin.. Chest.

