# Correction: Analysis of Pulmonary Inflammation and Function in the Mouse and Baboon after Exposure to *Mycoplasma pneumoniae* CARDS Toxin

**DOI:** 10.1371/annotation/616385db-f413-4f23-ba78-2fe626870e46

**Published:** 2009-12-11

**Authors:** R. Doug Hardy, Jacqueline J. Coalson, Jay Peters, Adriana Chaparro, Chonnamet Techasaensiri, Luis D. Giavedoni, Angelene M. Cantwell, T. R. Kannan, Joel B. Baseman, Peter H. Dube

Dr. Luis D. Giavedoni was not included in the author byline. He should be listed as the sixth author and affiliated with Department of Virology and Immunology and Southwest National Primate Research Center, Southwest Foundation for Biomedical Research, San Antonio, Texas, United States of America.

The author contributions should also be amended to the following: Conceived and designed the experiments: TRK JBB RDH JP PD. Performed the Experiments: RDH JC JP AC CT AC TRK LDG. Analyzed the data: RDH JC TRK AC LDG CT AC JBB PD. Contributed reagents/materials/ analysis tools: AC TRK JBB. Wrote the paper: JC JBB PD.

